# Anti-HTLV-1/2 IgG Antibodies in the Breastmilk of Seropositive Mothers

**DOI:** 10.3390/microorganisms9071413

**Published:** 2021-06-30

**Authors:** Carolina Rosadas, Timothy Woo, Jana Haddow, Aileen Rowan, Graham P. Taylor

**Affiliations:** 1 Department of Infectious Disease, Imperial College London, London W2 1PG, UK; timothy.woo18@imperial.ac.uk (T.W.); a.rowan@imperial.ac.uk (A.R.); 2 National Centre of Human Retrovirology, Imperial College Healthcare NHS Trust, London W21 NY, UK; jana.haddow@nhs.net

**Keywords:** HTLV-1, HTLV-2, mother-to-child transmission, vertical transmission, antibodies, breastmilk

## Abstract

Background: HTLV-1/2 mother-to-child transmission (MTCT) is an important route for the maintenance of HTLV-1/2 within populations and disproportionally contributes to the burden of HTLV-1-associated diseases. Avoidance of breastfeeding is the safest recommendation to prevent MTCT. Due to the benefits of breastfeeding, alternative methods that would allow seropositive mothers to breastfeed their babies are needed. There is limited knowledge about HTLV-1/2 infection and breastmilk. Methods: Paired blood and milk samples collected from HTLV-1/2 seropositive mothers were tested for HTLV-1 proviral load (PVL) quantification and for the detection of anti-HTLV-1/2 IgG. Results: All breastmilk samples had detectable anti-HTLV-1/2 IgG. HTLV-1/2 proviral DNA was detected in all samples except for one. HTLV-1 PVL and IgG binding ratio (BR) was similar in milk and plasma. However, antibody titer was significantly higher in blood (Median (95%CI): Milk:128 (32–512); Plasma:131,584 (16,000–131,584), *p* < 0.05). There was a strong correlation between HTLV-1 PVL, anti-HTLV-1/2 IgG BR, and titer when comparing milk and blood. PVL did not correlate with antibody BR nor titer in blood or milk. Conclusions: Anti-HTLV-1/2 IgG are present in milk in the same proportion as blood but in lower quantity. PVL in milk correlates with blood.

## 1. Introduction

Human T-cell lymphotropic virus (HTLV) infects at least 5–10 million individuals in the world [1]. HTLV-1 causes a range of diseases, including a severe leukaemia, called adult T-cell leukaemia/lymphoma (ATLL), and a debilitating and progressive neurological disease known as HTLV-1-associated myelopathy (HAM). It is becoming increasingly evident that the impact of HTLV-1 is wider still and a multitude of inflammatory conditions including uveitis, infective dermatitis, and lung inflammation may also be caused by this virus [2] and this may, in part, explain the 57% increase in the adjusted mortality rate associated with HTLV-1 infection [2]. HTLV-2 is rarely associated with disease and is less prevalent than HTLV-1. These retroviruses maintain life-long infection mainly by clonal expansion of infected cells. Virions are rarely produced in stable chronic infection [3]. Therefore, the transmission of this virus occurs by contact with infected cells that are present in different bodily fluids such as blood, seminal and vaginal fluids, and breastmilk.

In HTLV mother-to-child transmission (MTCT), infected lymphocytes carrying integrated HTLV-1 proviruses are transferred from mother to child either in utero, during delivery, or through the transfer of infected cells during breast feeding [4]. Ante-natal transmission is considered to be rare, as only 2.5% of exclusively formula-fed children born from seropositive women will become infected. On the other hand, HTLV-1 transmission occurs in up to 32% of infants that are breast-fed by seropositive mothers [4]. The higher rates of transmission through prolonged breastfeeding are thought to be associated with declining levels of transplacental anti-HTLV-1 antibodies in infants over time and cumulative exposure to HTLV-1.

The main recommendation to avoid MTCT is the suspension of breastfeeding and provision of formula milk to those babies born from seropositive mothers [5]. However, as the benefits of breastfeeding are extensive, a better understanding of the mechanism behind oral transmission of HTLV-1/2 and protective pathways is crucial for the development of alternative measures to avoid vertical transmission. This is considered a priority according to the World Health Organization (WHO), as stated in the recently published HTLV Technical Report [6]. Indeed, mother-to-child transmission is associated with higher risk of HTLV-1-associated diseases and this route of infection facilitates the maintenance of silent transmission within families and populations [4].

Previous studies have not detected anti-HTLV-1 antibodies in milk [7,8,9]. Here, the presence of anti-HTLV antibodies in breastmilk from seropositive mothers was assessed using a newly developed anti-HTLV IgG capture assay that is suitable for the detection of antibodies in bodily fluids [10]. HTLV-1/2 proviral load and anti-HTLV-1/2 antibodies in milk and plasma were also compared.

## 2. Materials and Methods

Nine paired breastmilk and EDTA blood samples were collected from seven women with HTLV-1/2 asymptomatic infection attending the National Centre for Human Retrovirology, London, UK during post-natal follow-up. One was infected by HTLV-2 and six with HTLV-1. One woman had samples available from post-partum periods of two pregnancies and another woman donated samples in two different time points after a single delivery. An additional pair of samples obtained from a woman not infected by HTLV-1 was included as a negative control. Plasma and peripheral blood mononuclear cells (PBMCs) were separated by Ficoll density gradient centrifugation. Breastmilk samples were collected by manual expression and cells were obtained by centrifugation. HTLV proviral load (PVL) was quantified in peripheral blood mononuclear cells (PBMCs) and breastmilk cells by real-time PCR [11], while plasma and breastmilk supernatant were used for antibody detection. Samples with undetectable PVL by real-time PCR were further tested using a highly sensitive nested PCR [12].

Anti-HTLV-1/2 antibodies were detected using the anti-IgG capture immune assay, as previously described [10]. Briefly, 100 uL of samples (undiluted breastmilk or plasma diluted in 1:200) were added to wells coated with anti-human IgG (5 μg/mL). After incubation at 37 °C for 1 h, the wells were washed and HTLV-1/2 proteins conjugated with horseradish peroxidase were added (HTLV-1/2 Murex Kit). The wells were incubated for 2 h (37 °C) and a subsequent wash was performed. The reaction was revealed using TMB and stopped with Sulphuric Acid after 30 min incubation. The optical density (OD) of each well was determined using a spectrophotometer (Spectramax) at 450 nm. A negative sample was tested in triplicate in each run. The cut-off was calculated by adding 0.1 to the average of the OD of negative samples. The binding ratio (BR) was determined dividing the OD of each sample by the cut-off value. BR ≥ 1 was considered positive. Titration of seropositive samples was performed by serial dilution (1:2). A sample’s titer is defined as the highest dilution that still yields a positive reading.

Wilcoxon paired test was used to compare IgG BR, IgG titer, and HTLV PVL between plasma and breastmilk samples. The correlation between variables (BR, Titer, and PVL) in plasma and breastmilk was assessed using Spearman’s test. The same test was used to identify if the BR and antibody titer correlated to HTLV-1 PVL.

All samples were donated to the Communicable Diseases Research Group Tissue Bank (UK National Research Ethics Service Reference 20/SC/0029) after participants had provided written informed consent.

## 3. Results

**Figure 1:**
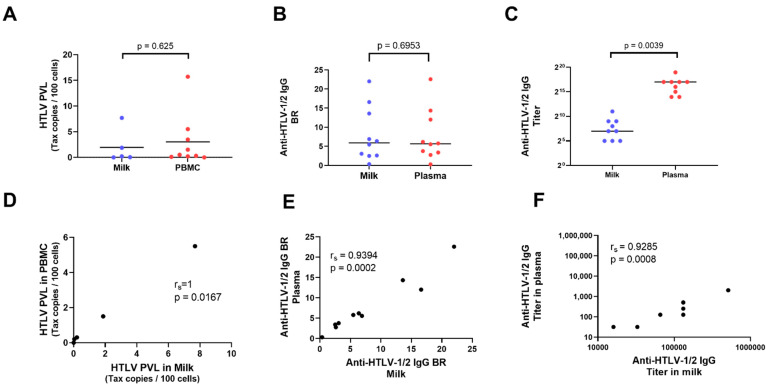
Anti-HTLV-1/2 IgG and proviral load in plasma and milk samples. (**A**) Comparison of anti-HTLV-1/2 proviral load (PVL) in milk and plasma measured by real-time PCR. (**B**) Comparison of anti-HTLV-1/2 IgG binding ratio (BR) in milk and plasma using anti-HTLV-1/2 IgG capture ELISA assay. (**C**) Comparison of anti-HTLV-1/2 IgG titer in milk and plasma serially diluted in 1:2. *Y* axis is in log2 scale. Each dot represents a sample. Horizontal bars represent median. Wilcoxon paired test was used to compare groups and *p* values are shown. (**D**) Correlation between HTLV-1/2 PVL in peripheral blood mononuclear cells (PBMCs) and milk. (**E**) Correlation between anti-HTLV-1/2 IgG BR in plasma and milk. (**F**) Correlation between anti-HTLV-1/2 IgG titer in plasma and milk. Each dot represents a sample. Correlation coefficient (Spearman test) and *p* values are shown.

**Figure 2:**
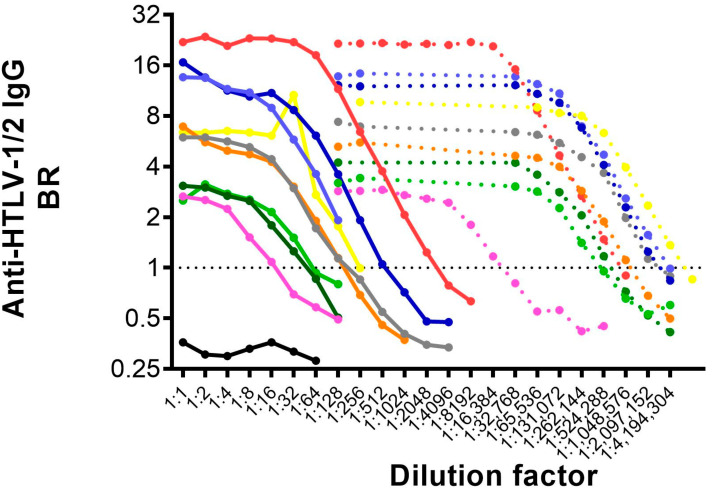
Titration of anti-HTLV-1/2 IgG in paired plasma and milk samples. Paired plasma and blood samples were serial diluted in crystalloid buffer and tested in the anti-HTLV-1/2 IgG capture ELISA assay. Horizontal black dashed line shows the assay cut-off. Plasma samples are represented by a dashed line and milk samples by a continuous line. Each individual is represented by a different color. Light and dark green shows samples from the same patient collected after distinct pregnancies. Light and dark blue are samples from the same patient collected at different time-points after the same pregnancy. A black, continuous line shows the negative control.

All breastmilk samples had detectable anti-HTLV-1/2 IgG. There was no significant difference between the BR of anti-HTLV-1/2 IgG between blood plasma and breast milk supernatant (Median (95%CI): Milk: 5.9 (2.5–16.6); Plasma: 5.7 (2.8–14.4), *p* > 0.05). However, the antibody titer was significantly higher in blood (Median (95%CI): Milk:128 (32–512); Plasma:131,584 (16,000–131,584) (Figure 1B, C). Anti-HTLV-1/2 IgG titer ranged between 32 and 2048 in milk and from 16,448 to 524,288 and blood (Table 1 and Figure 2).

HTLV-1/2 proviral load is presented as the number of HTLV DNA molecules per 100 cells and abbreviated to %. HTLV-1/2 DNA was detected in all samples available except for one in which HTLV-1 PVL in PBMCs was 0.002/ 100 PBMCs (Table 1). HTLV-1 proviral load was similar in milk and plasma (Median (95%CI): Milk: 0.2 (0–7.7); Plasma: 0.5 (0.1–5.5), *p* = 0.625) (Figure 1A).

There was a perfect correlation between HTLV-1 proviral load in milk and PBMC (Figure 1D). A strong correlation was also observed between anti-HTLV-1/2 IgG BR in milk and plasma (Figure 1E), and between the anti-HTLV antibody titer in these two fluids (Figure 1F).

**Figure 3:**
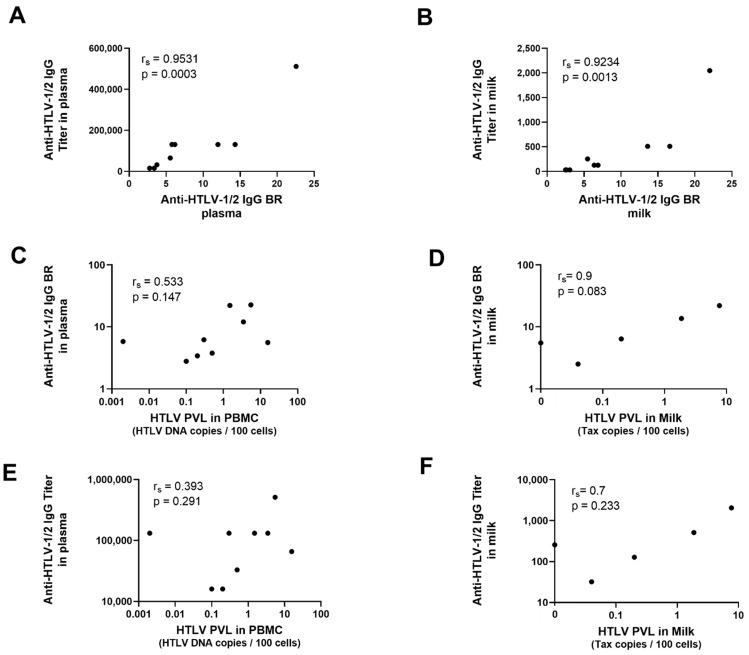
Correlation between HTLV-1/2 proviral load and anti-HTLV-1/2 IgG antibodies. Milk and plasma samples were tested using real-time PCR for the determination of HTLV-1/2 proviral load and with anti-HTLV-1/2 IgG capture assay for the determination of the binding ratio (BR) and the antibody titer. Correlation coefficient was calculated using Spearman test. The results are shown with their respective *p* value. Correlation between anti-HTLV-1/2 IgG BR and titer in blood (**A**) and in milk (**B**); Correlation between anti-HTLV-1/2 IgG BR and HTLV-1/2 PVL in blood (**C**) and in milk (**D**); Correlation between anti-HTLV-1/2 IgG Titer and HTLV-1/2 PVL in blood (**E**) and in milk (**F**).

Anti-HTLV-1/2 IgG BR correlated with antibody titer (Figure 3A,B). The PVL did not have a statistically significant correlation with antibody BR nor titer in blood or milk (Figure 3C,D).

The only patient who had samples collected after two pregnancies had comparable results, as well as the patient who had samples available from two different time points after delivery (Table 1, Figure 2).

## 4. Discussion

Understanding the mechanism behind mother-to-child transmission and protection is essential to develop alternatives to prevent vertical transmission of HTLV-1/2 and the burden of the diseases associated with this virus. This study showed that anti-HTLV-1/2 IgG was present in the milk of seropositive mothers. As IgG capture assay is a proportionality assay: its BR is associated with the proportion of target antibodies in the tested sample, rather than in their quantity [10]. Although this characteristic is important to increase the assay sensitivity when assaying bodily fluids, the BR is not a measure of quantity of target antibodies. In fact, while there was no difference between the BR observed in milk and plasma, anti-HTLV-1/2 IgG titer was approximately 1000 times higher in plasma than in milk. Therefore, although anti-HTLV-1/2 antibodies were present in the milk in the same proportion as observed in plasma, the quantity was lower.

**Figure 4:**
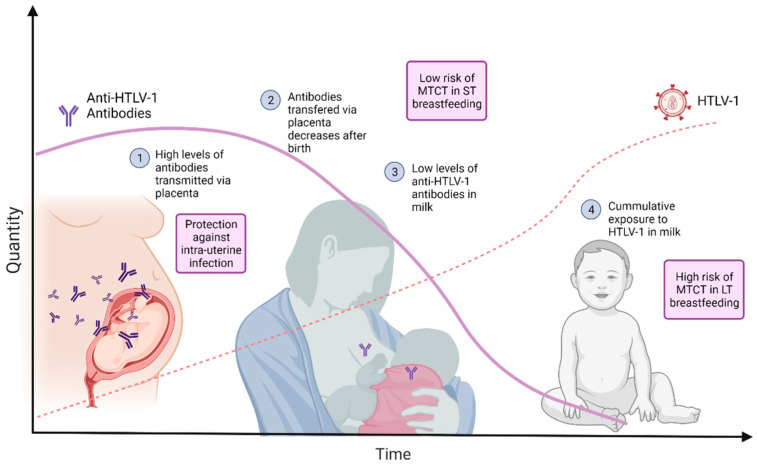
Graphic illustration of the hypothesis for higher risk of HTLV-1/2 infection with long-term breastfeeding. High levels of anti-HTLV-1 antibodies are transferred via placenta during pregnancy. These antibodies contribute to protection against intra-uterine infection. After birth, those antibodies passively transferred in-utero decrease. The level of antibodies in milk is low. As HTLV-1 is present in breast milk, there is a cumulative exposure to HTLV-1. The hypothesis is that this will culminate in increasing risk of HTLV-1 transmission with long-term breastfeeding. Image Created with BioRender.com in June 2021.

Our group showed previously that pregnant women infected by HTLV-1 had a discrete but statistically significant increase in anti-HTLV-1/2 antibody levels during pregnancy [13] and, although in that occasion no difference was found in HTLV PVL during pregnancy [13], there is a report of increased HTLV PVL in the blood following delivery [14]. This is in line with the hypothesis that a high number of antibodies are transplacentally transmitted during pregnancy and may have a protective effect. Thus, an increase in maternal antibodies during pregnancy might play an important role in preventing intra-uterine transmission and also perinatal transmission through the passive transfer of antibodies via the placenta. This would at least in part explain the lower levels of transmission rates observed during pregnancy even though placental villous tissues of the foetuses of nearly half of pregnant carriers are infected by HTLV-1/2 [15]. In fact, the hemochorial placenta allows the transfer of high rates of IgG before birth. At delivery, neonates can carry even higher titers of IgG compared with their mothers [16]. This may account for the low risk of MTCT in the context of short-term breastfeeding. In contrast, maternal transfer of IgG after birth, via milk, is considered to be low in humans, either due to low levels of IgG in milk or due to low rates of gastro-intestinal IgG absorption [17]. Therefore, as breastfeeding persists, there is a cumulative exposure to HTLV-1/2, while the passively transferred maternal antibodies decline and the amount of antibody in the milk, as demonstrated here, is low, increasing the risk of infection (Figure 4). This observation highlights the potential role of antibodies in the protection of vertical transmission. Indeed, antibodies are routinely used in clinical practice to prevent other viral infections, such as the vertical transmission of HBV.

In the two sequential samples included in this study, the titer of antibodies and PVL were very consistent. Whilst is difficult to extrapolate data on two cases to all lactating HTLV-1 infected mothers, these data did suggest that the quantity of antibodies and PVL may be stable during the post-partum period. Our group previously demonstrated the stability of HTLV proviral load, within individuals who were neither pregnant nor lactating, in established infection, over a median of eight years follow-up [11].

The detection of IgG in milk may be useful for the screening of donors in milk banks. A previous study showed the detection of antibody in milk donors; however, the HTLV-1/2 serostatus was unknown [18]. HTLV screening of milk donors is recommended in the UK and in France, being in the latter restricted to those donors from endemic regions [6].

This study also confirmed that there is a strong correlation between HTLV-1/2 PVL in blood and milk [19]. This indicates that the quantification of PVL in PBMCs, which is usually done in clinical routine, may be used to predict PVL in milk. There is no data on a threshold for PVL that may stratify the risk of transmission through breastfeeding. The identification of such a threshold is recommended by the WHO, as it could potentially allow a proportion of women living with HTLV-1/2 to breastfeed their babies safely [6].

This study had some limitations such as the small number of samples, the fact that all patients had asymptomatic infection, and most of them were breastfeeding for a short period due to the local recommendation for seropositive mothers to avoid or limit short-term breastfeeding.

For those who wish to breastfeed, the risk versus benefit should be balanced on an individual basis, considering: the viral type, as HTLV-2 is less pathogenic than HTLV-1; risk factors that may contribute to MTCT, such as HTLV-1/2 PVL and the presence of *Strongyloides* sp. co-infection; and access to safe, affordable formula feeding. A recent meta-analysis showed that short-term breastfeeding was not associated with a significant increase in the transmission of HTLV-1 [20]. However, the data were mainly restricted to Japan and may not be representative in other settings. In addition, 8–18% of those mothers who opted for short-term breast-feeding continued for a longer period than recommended [21]. Freeze-and-thaw breastmilk can also be used to decrease the risk of transmission for those mothers that are willing to breastfeed [22,23]. Although likely to be effective, there are no data on pasteurization for HTLV-1. At present, the most effective way to prevent HTLV-1 MTCT is to avoid breastfeeding along with provision of formula milk where this can be safely offered to HTLV-1-seropositive women.

## Figures and Tables

**Table 1:** HTLV-1/2 proviral load and anti-HTLV-1/2 IgG in blood and milk.

Sample ID	Age (Years)	Time Since Delivery (Days)	Blood			Milk		
			PVL	BR	Titer	PVL	BR	Titer
1	37	92	5.5	22.58	524,288	7.69	22	2048
2	27	71	0.3	6.17	131,584	0.2	6.38	128
3	26	58	0.002	5.79	131,584	0	5.48	256
4 *	32	14	0.1	2.77	16,448	NA	2.62	32
5	28	456	15.7	5.56	65,792	NA	6.9	128
6 ^a^	33	11	0.5	3.75	32,896	NA	3.07	32
7 ^b^	36	17	0.2	3.4	16,448	0.04	2.5	32
8 ^#^	49	8	1.5	14.33	131,584	1.87	13.6	512
9 ^#^	49	52	3.48	12	131,584	NA	17	512
Neg.	35	111	Neg.	0.31	Neg.	Neg.	0.36	Neg.

PVL: HTLV-1/2 proviral load; BR: Binding ratio; * HTLV-2 patient; ^a,b^ same patient in different pregnancy; # same patient same pregnancy.

## Data Availability

All data are included in the manuscript.
